# Family religiosity and climate: the protective role of personal interiorized religiosity in deviance propensity among justice-involved juveniles

**DOI:** 10.3389/fpsyg.2024.1197975

**Published:** 2024-04-29

**Authors:** Valeria Saladino, Oriana Mosca, Cristina Cabras, Valeria Verrastro, Marco Lauriola

**Affiliations:** ^1^Department of Human, Social and Health Sciences, University of Cassino and Southern Lazio, Cassino, Italy; ^2^Department of Education, Psychology, Philosophy, University of Cagliari, Cagliari, Italy; ^3^Department of Health Sciences, University of “Magna Graecia”, Catanzaro, Italy; ^4^Department of Developmental and Social Psychology, University of Rome “Sapienza”, Rome, Italy

**Keywords:** justice-involved juveniles, religiosity, family, deviance propensity, new development goals

## Abstract

According to the literature, religious commitment could be a protective factor against dangerous behaviors, such as criminal offending, unsafe sex, and substance use. Our study aims to investigate the influence of Family Religiosity and climate on anger dysregulation and deviance propensity in a sample of 214 justice-involved boys from Italian Youth Detention Centers (range 14–25). The sample was divided into religious (*n* = 102) and non-religious (*n* = 112) justice-involved juveniles. Participants filled in the following questionnaires: Deviant Behavior Questionnaire, Aggression Questionnaire, Family Communication Scale, Moral Disengagement Scale, and the Multidimensional Scale of Perceived Social Support. We used a partial least square structural equation modeling (PLS_SEM) method to build our model and we found that Family Religiosity was positively associated with Family Climate which was negatively associated with Anger Dysregulation and Deviance Propensity, and Anger Dysregulation was positively related to Deviance Propensity. The multigroup analysis confirmed that for justice-involved juveniles who interiorized religious discipline and beliefs, Family Religiosity showed a positive association with Family Climate, which had a negative relationship with Anger Dysregulation, which strongly predicted Deviance Propensity. This result could be useful to promote new development goals and preventive activities and interventions based on positive religiosity values in juveniles’ behavior.

## 1 Introduction

The religion–crime association originates in the earliest criminological writings; however, it was not until the publishing of Hirschi and Stark’s “Hellfire and Delinquency” study (Hirschi and Stark, 1969) that this relationship became a noticeably larger area of study in the criminological literature, despite the null findings highlighted in this seminal work (Watts, 2018). Since then, the empirical literature on the religion–crime relationship has continued, with many studies finding a protective effect of religiosity. Subsequent research investigating the link between religiosity, deviant behavior, and crime has shown that religious involvement and belief often function as a moderate deterrent to criminal behavior in multiple domains (Baier and Wright, 2001; Salas-Wright et al., 2015). This buffer effect seems to function in a unidirectional way and with an inverse relationship so that higher levels of religiosity generally are associated with lower levels of criminal behavior (Salas-Wright et al., 2015). One of the most convincing examples of the protective effect of religiosity was the meta-analysis of 60 research articles conducted by Baier and Wright (2001) that looked at the religiosity–crime relationship. The authors found that across multiple studies and using various measures of religiosity, the latter consistently exhibited a significant, negative relationship with engagement in various criminal behaviors. Another even more recent analysis of the religiosity-crime literature that looked at 270 articles published between 1944 and 2010 found similar results, with more than 90% of the articles showing that religiosity was a deterrent against crime and other antisocial behaviors (Johnson and Jang, 2010). Most of the research conducted thus far has generally indicated that religion exerts a protective effect against crime, with some disagreement arising regarding the specific circumstances (e.g., when and how) in which religion serves as a protective factor against deviant behavior. There is an extensive literature concerning direct relationships between religiosity and crime. However, research about moderators of the religiosity–crime relationship is scarce (Watts, 2018), even though there are identifiable differences in religious engagement across many factors, such as sex and age, and private versus public religiosity (Salas-Wright et al., 2015).

### 1.1 Personal religiosity and criminal behavior among juveniles

The concept of religiosity is comprehensive of all dimensions of religion, from rituals and beliefs to social traditions and familial practices (Gallagher and Tierney, 2013). Several studies have investigated the role of religion on juvenile delinquency, showing an inverse relationship between religiosity and crime. According to the literature, higher levels of religious participation can be considered a protective factor against antisocial behaviors, like criminal activities, substance abuse, and other at-risk behaviors (DeCamp and Smith, 2019; Jang, 2019; Pardini et al., 2020). Indeed, juveniles with personal religiosity tend to engage in less violent and criminal behavior (Salas-Wright et al., 2015; Salvatore and Rubin, 2018).

Personal religiosity could be influenced by other factors, such as demographic characteristics, that can mediate or moderate the role of religion as a protective element. Furthermore, research also indicated that religion does not directly affect criminal offending (Guo, 2020). According to these results, a spurious relationship between religiosity and crime could be hypothesized, considering the variables used for examining the construct of religion. Due to these controversial results, the aspects of religion that are directly involved in juvenile delinquency and the mechanisms responsible for crime reduction, need to be clarified (*Ibidem*).

Sumter et al. (2018) have claimed that some aspects of religion are directly responsible for reducing participation in criminal activities, such as religious beliefs associated with self-control and social control (Hirschi, 1969). Current evidence indicated that higher levels of social control and religion could reduce criminal offending (Syahrivar et al., 2022). To explain the protective role of religion, past research has focused on several popular theories in criminology, including self-control theory (Salas-Wright et al., 2015), social bonds theory (Chu, 2007), and social learning theory (Adamczyk, 2012). According to the self-control theory, self-control is the capacity of individuals to intentionally refrain from involvement in immediately gratifying behaviors based on subsequent expected benefit or conformity with social or moral expectations (Hirschi and Gottfredson, 1993). According to these authors, four types of social bonds influence criminal conduct: attachment, commitment, involvement, and belief. Individuals with high levels of self-control are less likely to engage in deviant behavior or to be influenced by a criminogenic background.

Attachment was considered by the author an influential factor. Individuals with poor social bonds are more likely to engage in criminal and risky behaviors, especially during adolescence. Also, individuals who show secure attachment are less likely to commit crimes during adolescence and adulthood than others (Saladino et al., 2021). Along the same line, the reconstruction of social bonds and establishing connection to others after incarceration have a protective role in the life course of people previously incarcerated, decreasing the recidivism risk (Saladino et al., 2020).

Authors identified as commitment the degree of devotion to traditional ideals and objectives. Hirschi assumes that someone who has already committed resources and efforts to reaching conforming goals stands to lose more from deviant behavior than someone who has not given much thought to seeking socially acceptable goals (Krohn and Massey, 1980).

Similarly, Hirschi uses the term involvement to describe how someone who participates in traditional activities has less time and opportunities to engage in deviant conduct, showing more self-discipline (Agnew, 1985).

Finally, Hirschi describes beliefs as values and norms. According to the author, the more the individuals internalized values, the more difficult it becomes to violate them. All these factors could be related to religiosity. Indeed, frequent attendance of religious activities seems to be related to high levels of self-control, reducing the propensity for deviant behavior. Also, religiosity creates social bonds inside and outside the family system (Charles et al., 2020). Religious activities guarantee a sense of commitment and involvement with the community and the family, reducing the risk of being involved in criminal conduct. Specifically, the internationalization of religious beliefs could be a protective factor in developing deviant trajectories thanks to its association with prosociality and empathy (Tsang et al., 2021).

Involvement in religious activities increases the feeling of disapproval for deviant behaviors, reducing the interaction with criminal peer groups. Indeed, being part of a religious community can influence people’s behavior. For instance, committed religiosity seems to positively impact desistance from deviant behaviors, such as sexual crimes (Harris et al., 2017; Stansfield, 2018). This positive association is also linked to social connectedness and emotional regulation, which influence personal religiosity and interpersonal relationships with others (Vishkin et al., 2019). In a recent theoretical development concerning social bonding and self-control theories, Hirschi (2004) has proposed a slight revision of the self-control construct, essentially arguing that “social control and self-control are the same thing” (see also Hirschi and Gottfredson, 2006). Self-control is then redefined as, “the set of inhibitions one carries with one wherever one happens to go” (Hirschi, 2004, pp. 5435,44). In this revised version of self-control theory, the nature of these inhibitions, one’s level of self-control at any one time, can be described through the elements of the social bond for that individual: attachment, commitments, involvement, and beliefs (Hirschi, 2004). With this new definition of self-control, religiosity can be thought of as both a social bond and an element of self-control within one’s life (Watts, 2018).

### 1.2 Familial religiosity and criminal behavior among juveniles

Parenting and family participation in social activities influence the development of personal religiosity among individuals. Generally, parental supervision and family attachment are considered protective factors for young criminality. Spencer (2014) has shown that family participation in religious activities is negatively associated with juvenile delinquency. The relationship between familial religiosity and youth delinquent behavior was found to be mediated by the marital relationship, parental monitoring, and parents’ attachment (Bowlby, 1982; Ainsworth, 1985; Pickering and Vazsonyi, 2010; Guo, 2018). The children-parent relationship is the context for developing emotion regulation, social skills, and learning family values, including the ones linked to the religious domain.

According to Bowlby’s theory, attachment and caregiving guarantee positive development (Bowlby, 1969; Van Rosmalen et al., 2016). The attachment style affects the capacity to engage in social relationships during adulthood. Thus, justice-involved juveniles could use criminality as a problem-solving strategy (De Li, 2004; Chapple, 2005).

Individuals who have a secure attachment tend to develop social skills and deal successfully with stressful situations; while insecure attachment and poor parenting can increase antisocial behaviors and criminality (Ryan and Testa, 2005). Safe attachment style and close relationships with parents decrease the rate of bullying and violence (Cho et al., 2017) and promote relational and social skills (Groh et al., 2017). Adolescents with secure attachment have more confidence and better emotional regulation skills than adolescents with avoidant attachment, who show more difficulty in interpersonal relationships (Clear and Zimmer-Gembeck, 2017).

Moreover, the sense of belonging to the family or surrogates like school, peers, and religious communities mediate the use of violence during adolescence, guarantees informal control during the transition to adulthood and reduces the likelihood of future involvement in the legal system (Saladino et al., 2020).

Attachment theory could be applied to religiosity considering the relationship with God (D’Urso et al., 2019). Believers who establish a secure attachment to God tend to develop safe and reliable relationships in other domains. A cognitive schema of God’s image seems to affect people’s attitudes and behaviors, including crime and deviance (Cabras et al., 2019; Barberis et al., 2022; Cabras et al., 2022). Jang et al. (2018) found that religiosity and belief in God’s engagement and forgiveness are inversely related to violent acts among justice-involved people. Indeed, imagining a caring and forgiving God impacts offenders’ behavior positively, promoting the development of prosociality.

This image is often related to parental beliefs on religiosity. Religious parents seem more likely to have a positive marital relationship, good communication, and support with an improved quality of family life that contributes to the development of youth self-control (Mafi and Havassi, 2023). Overall, parental religiosity can decrease family conflict and improve parent–child relationships that facilitate social bonds and reduce delinquent involvement (Goeke-Morey and Cummings, 2017). Furthermore, parental religiosity could operate as a social influence on the religious and spiritual life of juveniles. Finally, the literature underlined an interrelation between family dynamics and juvenile delinquency (Saladino et al., 2021) without considering family religiousness and interiorized religiosity as protective factors for deviancy.

### 1.3 Aim of the study

The theoretical framework for this study is grounded in the empirical research and theory on the relationships between self-control and deviant behavior in the developmental process. To address the existing gap in the literature, our research aims to study the influence of Family Religiosity and climate on Anger dysregulation and Deviance propensity (the variables are described in detail in the 2.3. Data Analysis section). We compared the hypothesized relationships in two groups: religious vs. non-religious justice-involved juveniles from Italian Youth Detention Centers. We expect that:

*H1*: Family Religiosity and positive Family Climate could function as buffers for Anger Dysregulation and Deviance Propensity in all justice-involved juveniles;

*H2*: The hypothesized relationships are moderated by participants’ religiosity: the religious justice-involved juveniles are less likely to develop Anger Dysregulation and Deviance Propensity.

## 2 Materials and methods

### 2.1 Participants

Participants involved were 214 male justice-involved juveniles (33.17% foreigners) from Italian Youth Detention Centers (mean age: 19.02 years old) (sd = 2.20; range 14–25). The sample was divided into religious (*n* = 102) and non-religious (*n* = 112) justice-involved juveniles. Researchers explained the study goals to potential participants and obtained informed consent. The inclusion criteria were the following: juveniles from 14 to 25 years old and a good command of the Italian language. The protocol applied also to those who are foreign accompanied by a cultural mediator. The study was conducted following the Declaration of Helsinki, was approved on October 9, 2019, by the Institutional Review Board of the University of Cassino and Southern Lazio (Italy) and evaluated by the Ministry of Justice and the Directors of the involved Italian Youth Detention Centers.

### 2.2 Measures

#### 2.2.1 Deviant behavior questionnaire

The Deviant Behavior Questionnaire (DBQ) measures adolescents’ common risky and deviant behaviors and was adapted from the international self-reported delinquency study (Enzmann et al., 2015). The DBQ is composed of 9 items with dichotomous format answers (yes/no) measuring the tendency to commit illegal actions (e.g., “Have you ever illegally downloaded music or movies?”; “Have you ever stolen anything?”) and to show aggressive attitudes (e.g., “Have you ever threatened or assaulted someone with a weapon?”; “Have you ever verbally or physically attacked someone?”). The internal consistency coefficient in the present study is 0.79.

#### 2.2.2 Aggression questionnaire

The Aggression Questionnaire (AQ, Buss and Perry, 1992; Sommantico et al., 2015) is composed of 29 items measuring the tendency to commit aggressive behaviors on a Likert scale from 1 to 5 (1 = totally false; 5 = totally true). The AQ is composed of four factors: Physical Aggression (PA) (e.g., “I’ve been so angry that I destroy things”), Verbal Aggression (VA) (e.g., “I openly tell my friends when I disagree with them”), Anger (A) “When I’m frustrated, I openly show my irritation” and Hostility (H) “I know that my “friends” talk about me behind my back.” Internal consistency coefficients in the present study are the following: Anger (0.68), Physical Aggression (0.79), Verbal aggression (0.52), and Hostility (0.62).

#### 2.2.3 Family communication scale

The Family Communication Scale (SCF, Barnes and Olson, 1982; Italian adaptation, D'Atena and Ardone, 1991) is a self-report questionnaire composed of 24 items, counted two times, separately for the mother and the father, evaluated on a Likert scale from 1 to 5 (1 = strongly disagree; 5 = strongly agree). Internal consistency coefficients of the scale in the present study are the following: Communication with mother (0.78) and Communication with father (0.80).

#### 2.2.4 Moral disengagement scale

The Moral Disengagement Scale (Pelton et al., 2004) is a self-report questionnaire that evaluates the homonymous construct identified by Bandura (1991), who described specific mechanisms created in response to violations of socially recognized moral values (e.g., displacement of responsibility, dehumanization of the victim, attribution of blame to the victim, distortion of consequences). As the moral disengagement increases, the sense of guilt and the need for repair decrease (e.g., “It is good to use force against those who offend your family”; “Stealing some money is not at all serious compared to those who steal large amounts of money”; “Mocking does not hurt anyone,” “Some people deserve to be treated harshly because they do not have feelings that can be hurt”). In its version for adolescents, the scale consists of 14 items evaluated on a Likert scale from 1 to 5 (1 = completely false; 5 = completely true). The higher the score is reported, the higher the moral disengagement. The internal consistency coefficient for the scale in the present study is 0.85.

#### 2.2.5 Multidimensional scale of perceived social support

The Multidimensional Scale of Perceived Social Support (Prezza and Principato, 2002) consists of 12 items that are grouped into three factors: Family, Friends, and Significant Others. The respondents were asked to indicate their level of agreement with each item by using a seven-point Likert scale ranging from 1 “very strongly disagree” to 7 “very strongly agree.” Higher scores indicated higher perceived social support. For the aims of the present study, we only used the Family dimension. The internal consistency coefficient for the Family subscale in the present study is 0.82.

### 2.3 Data analysis

The analyses were carried out using Smart PLS 3 (Partial Least Squares Structural Equation Modeling, PLS-SEM) which is a path analysis method recommended when the goal of the study is model building rather than theory testing and the sample size is not large enough for Covariance Based Structural Equation Modeling (CB-SEM) (for details see Ringle et al., 2015; Hair et al., 2017). Moreover, PLS-SEM works well with non-normal, or skewed data, and needs no parametric assumptions (Hair et al., 2017). The model tested had two exogenous variables (Family Religiosity and Family Climate) and two endogenous variables (Anger Dysregulation and Deviance Propensity). Family Religiosity was measured using three indicators: if the participants’ family was religious, if the participants’ parents were religiously observant, and if the participants’ parents provided a religious education. These last questions were dichotomous ones with simple yes and no answers. Family Climate was measured by the Perceived Family Support score of the MSPSS and the composite scores of communications with the mother and communication with the father of the SCF (see 2.2. Measures). Anger Dysregulation was based on the AQ’s anger, hostility, and verbal aggression scores (see 2.2. Measures). Last, Deviance propensity was measured using a deviant behavior index of the DBQ, the MDS’s moral disengagement score, and the AQ’s physical aggression score (see also Saladino et al. (2020) for a similar analysis conducted on a large sample of Italian youths). In the sample, there were 102 and 112 justice-involved juveniles who defined themselves as religious and atheist, respectively. Whether or not the participants defined themselves as religious was used as a between-subject factor in multigroup analyses. As such, personal religiosity served as a moderator factor in the multi-group analysis. We evaluated the model according to established criteria for PLS-SEM (Hair et al., 2017). First, we assessed the measurement model and then the structural model. The former deals with the relationships between the empirical indicators and the latent variables, and the latter includes the direct and indirect relationships between latent variables. Four quality criteria determine the adequacy of the measurement model. Indicator reliability is achieved when all empirical indicators of a latent variable load are above 0.50. Construct reliability is supported when a latent variable’s Composite Reliability (CR) is above 0.60, or preferably above 0.70. The Average Variance Extracted (AVE) should be 0.50 or higher to support the convergent validity of the latent variables. Last, the discriminant validity of the latent variables is supported when the square root of the AVE is larger than the estimated correlations of that latent variable with other variables in the model (Fornell and Larcker, 1981). To evaluate the structural model, we calculated the determination coefficients (R^2^) for each endogenous latent variable, the predictive accuracy index Q^2^, and the significance of direct and indirect effects. According to Hair et al. (2017), R^2^ values of 0.75, 0.50, and 0.25 represent high, moderate, and small thresholds, respectively. Positive Q2 values indicate the model’s predictive relevance (*Ibid*). The significance of the direct path coefficients is tested using nonparametric confidence intervals obtained from 5,000 bootstrap resampling iterations (Streukens and Leroi-Werelds, 2016). Each path effect size was assessed using the f^2^ statistics, a measure of change in R^2^ when a specific path is omitted. Small, medium, and large effect sizes were 0.02, 0.15, and 0.35, respectively (Hair et al., 2017).

**Table 1:** Means, standard deviations, skewness, kurtosis, and zero-order correlations between variables (*n* = 214).

	Min	Max	M	SD	Sk	C	1	2	3	4	5	6	7	8	9
1. Communication with mother	2.38	5.00	3.72	0.55	−0.10	−0.37	1								
2. Communication with father	1.14	5.00	3.36	0.67	−0.05	0.22	0.45^**^	1							
3. Perceived family support	1.00	7.00	5.75	1.50	−1.48	1.57	0.38^**^	0.30^**^	1						
4. Anger	1.00	4.86	3.02	0.85	0.00	−0.59	−0.22^**^	−0.13	0.02	1					
5. Physical aggression	1.11	5.00	3.13	0.92	−0.11	−0.83	−0.21^**^	−0.09	0.05	0.67^**^	1				
6. Verbal aggression	1.00	5.00	3.31	0.81	−0.40	0.33	−0.11	−0.00	0.12	0.63^**^	0.69^**^	1			
7. Hostility	1.00	4.88	3.01	0.78	−0.28	−0.22	−0.15^*^	−0.10	−0.01	0.62^**^	0.51^**^	0.56^**^	1		
8. Deviant behavior	0.00	1.89	0.51	0.32	0.30	0.15	−0.08	−0.23^**^	0.00	0.33^**^	0.43^**^	0.28^**^	0.19^**^	1	
9. Moral disengagement	1.00	4.93	2.73	0.87	0.11	−0.59	−0.23^**^	−0.08	0.02	0.43^**^	0.52^**^	0.43^**^	0.44^**^	0.26^**^	1

*p < 0.05, **p < 0.01.

## 3 Results

### 3.1 Descriptive analysis and correlations

To assess the distribution of the study variables, and investigate any multicollinearity issue, we conducted a descriptive statistics analysis and examined the linear correlations (Pearson’s r) among the variables (Table 1). The results revealed that the variables demonstrated a tendency toward normal distribution and there was no evidence of multicollinearity (*r* < 0.70).

### 3.2 Partial least squares structural equation modeling (PLS-SEM)

#### 3.2.1 Measurement model

All indicators, except the family support score, loaded on the respective latent variable much above 0.50, attaining the standard for indicator reliability (Table 2). In no case, the indicator variables loaded more on another latent variable than on the latent variable they were supposed to measure. The composite reliability indexes were above the recommended threshold of 0.70 for all the latent variables in the model, ranging from 0.75 for Family Climate to 0.89 for Anger Dysregulation (construct reliability). The AVE index was acceptable for Family Climate (i.e., AVE > 0.50) and good for Family Religiosity, Anger Dysregulation, and Deviance Propensity, supporting the convergent validity of the latent variables. Regarding discriminant validity, the square roots of the AVE (in the diagonal of Table 2) were higher than the correlations of the latent variables with other latent variables in the model (Table 2). The analyses have shown that composite and indicator reliability, convergent, and discriminant validity of the constructs, were acceptable.

**Table 2:** Single-group analysis.

Indicators	FR	FC	AD	DP
Loadings & cross-loadings matrix				
Observant Parents	**0.80**	0.22	0.00	−0.05
Parents teaching	**0.84**	0.20	−0.09	−0.12
Religious Parents	**0.88**	0.21	−0.04	−0.10
Family Support	0.13	**0.43**	0.06	0.04
Communication with father	0.14	**0.71**	−0.08	−0.13
Communication with mother	0.25	**0.93**	−0.18	−0.23
Anger	−0.07	−0.20	**0.89**	0.64
Hostility	0.00	−0.14	**0.83**	0.52
Verbal Aggression	−0.06	−0.07	**0.86**	0.65
Deviant Behavior	−0.13	−0.14	0.31	**0.62**
Moral Disengagement	−0.02	−0.19	0.50	**0.77**
Physical Aggression	−0.11	−0.18	0.73	**0.90**
Composite Reliability (CR)	0.88	0.75	0.89	0.81
Average Variance Extracted (AVE)	0.71	0.52	0.73	0.60
Construct correlation matrix				
Family Religiosity (FR)	0.84			
Family Climate (FC)	0.25	0.72		
Anger Dysregulation (AD)	−0.05	−0.16	0.86	
Deviance Propensity (DP)	−0.11	−0.22	0.71	0.77

Measurement model assessment.

AVE, Average Variance Extracted; CR, composite reliability; FR, Family Religiosity; FC, Familiy Climate; AD, Anger Dysregulation; DP, Deviance Propensity.

Factor loadings in the cross-loading matrix are reported in bold; cross-loadings are in regular font. Underlined coefficients in the diagonal of the construct correlation matrix are the squared roots of AVE. Coefficients below the diagonal are correlations among the latent variables in the model.

#### 3.2.2 Structural model

**Figure 1:**
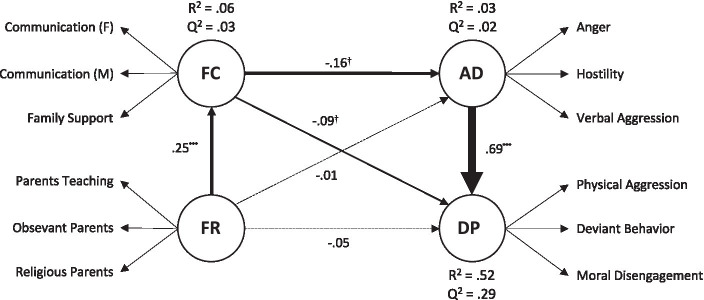
The estimated structural model. All paths were significant at *p* < 0.05. Line thickness is proportional to the size of the standardized regression coefficients. Nonsignificant regression paths have been omitted. FR, Family Religiosity, FC, Family Climate, AD, Anger Dysregulation, DP, Deviance Propensity.

The estimated structural coefficients, R^2^, and Q^2^ indexes for the endogenous variables are displayed in Figure 1. The model explained a moderately high proportion of Deviance Propensity variance. The effect sizes for Anger Dysregulation, and Family Climate, were small. The Q^2^ values were all positive, supporting the cross-validated predictive significance of the PLS path model. Family Religiosity was positively associated with Family Climate, with a small-medium effect size (f^2^ = 0.07). In turn, Family Climate was negatively associated with Anger Dysregulation and Deviance Propensity (both *p-s* < 0.10), but the effect sizes were small (both f^2^-s = 0.02). The most robust relationship was Anger Dysregulation with Deviance (f^2^ = 0.97). Next, we examined possible indirect relationships of Family Religiosity with Anger Dysregulation and Deviance Propensity. No indirect effect achieved the conventional levels of statistical significance. However, the analysis suggested that Family Religiosity might have an indirect relationship with Deviance Propensity but only when a one-tailed hypothesis test was used (Indirect = −0.03; 90% c.i. [−0.05; −0.00]).

### 3.3 Multigroup analysis

We hypothesized that the self-definition of religiosity among justice-involved juveniles could potentially impact model parameters and indirect effects. Accordingly, we tested the model separately for religious and nonreligious justice-involved juveniles, comparing the results between groups. Measurement model results are in Table 3. All indicators loaded on the respective latent variable above the standard threshold for indicator reliability in the religious group. For nonreligious justice-involved juveniles, an indicator of Family Climate (i.e., Communication with the father) failed to achieve the reliability standard. For both groups, all indicator variables loaded more on the latent variable they were supposed to measure than on another (Table 3). Reflecting the unsatisfactory performance of the Communication with the father indicator in the nonreligious group, the composite reliability for the Family Climate was below the threshold of 0.70. The remaining composite reliabilities were sufficient in both groups. Similarly, the AVE index was acceptable for all latent variables in both groups but for Family Climate for nonreligious justice-involved juveniles. Notwithstanding this, the square roots of the AVE were higher than the correlations of the latent variables with other latent variables in both groups (Table 2). The analyses have shown that composite and indicator reliability, convergent and discriminant validity of the constructs, were acceptable in both groups, except for Family Climate in nonreligious justice-involved juveniles.

**Table 3:** Multi-group analysis.

Indicators	Nonreligious justice-involved juveniles				Religious justice-involved juveniles			
	FR	FC	AD	DP	FR	FC	AD	DP
Loadings & cross-loadings matrix								
Observant Parents	**0.77**	0.11	0.08	0.10	**0.80**	0.23	0.01	−0.05
Parents teaching	**0.80**	0.15	−0.04	0.00	**0.87**	0.23	−0.02	−0.09
Religious Parents	**0.88**	0.15	−0.01	0.02	**0.81**	0.12	0.06	−0.08
Family Support	0.14	**0.98**	0.20	0.15	0.15	**0.55**	−0.08	−0.07
Communication with father	0.09	**0.27**	−0.03	−0.10	0.15	**0.71**	−0.09	−0.12
Communication with mother	0.21	**0.51**	−0.05	−0.08	0.24	**0.94**	−0.27	−0.35
Anger	0.07	0.13	**0.85**	0.59	−0.07	−0.26	**0.91**	0.69
Hostility	0.07	0.10	**0.81**	0.52	0.02	−0.21	**0.85**	0.53
Verbal Aggression	−0.08	0.19	**0.90**	0.69	0.10	−0.14	**0.82**	0.60
Deviant Behavior	−0.05	0.02	0.29	**0.50**	−0.06	−0.11	0.30	**0.66**
Moral Disengagement	0.11	0.10	0.45	**0.78**	−0.07	−0.33	0.54	**0.77**
Physical Aggression	0.02	0.12	0.73	**0.91**	−0.08	−0.23	0.72	**0.89**
Composite Reliability	0.86	0.65	0.89	0.78	0.86	0.79	0.89	0.82
Average Variance Extracted (AVE)	0.67	0.43	0.72	0.56	0.68	0.56	0.74	0.61
Construct correlation matrix								
Family Religiosity (FR)	0.82				0.83			
Family Climate (FC)	0.17	0.66			0.25	0.75		
Anger Dysregulation (AD)	0.01	0.17	0.85		0.01	−0.24	0.86	
Deviance Propensity (DP)	0.04	0.12	0.71	0.75	−0.09	−0.30	0.71	0.78

Measurement model assessment.

AVE, Average Variance Extracted; CR, composite reliability; FR, Family Religiosity; FC, Family Climate; AD, Anger Dysregulation; DP, Deviance Propensity.

Factor loadings in the cross-loading matrix are reported in bold; cross-loadings are in regular font. Underlined coefficients in the diagonal of the construct correlation matrix are the squared roots of AVE. Coefficients below the diagonal are correlations among the latent variables in the model.

**Figure 2:**
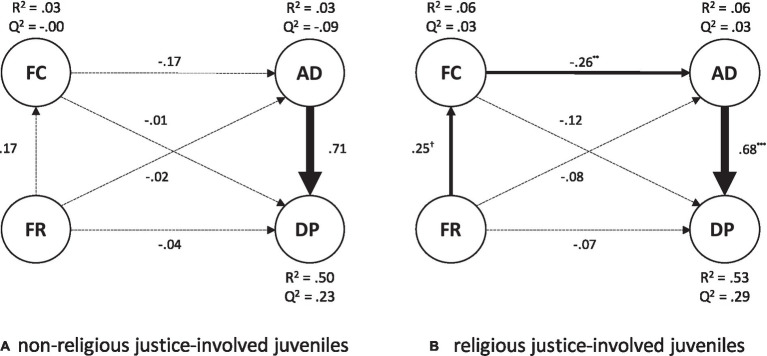
The estimated structural models in non-religious and religious justice-involved juveniles.

The structural coefficients, R^2^, and Q^2^ indexes for the endogenous variables estimated in each group are displayed in Figures 2A,B. In the nonreligious inmate group (Figure 2A), the only significant association linked Anger Dysregulation with Deviance Propensity with a considerable effect size (f^2^ = 0.98). As a result, the Q^2^ values were negative for Family Climate and Anger Dysregulation, showing that Family Religiosity was practically irrelevant in predicting these dependent variables. Conversely, the Q^2^ was positive for Deviance Propensity, but the link with Family Religiosity was null (f^2^ = 0.00). The analysis of religious justice-involved juveniles (Figure 2B) showed that the model accounted for a moderately high proportion of Deviance Propensity variance. The R^2^ for Anger Dysregulation, and Family Climate, were small. As in single-group analyses, the Q^2^ values were all positive, supporting the predictive significance of the path model. Likewise, Family Religiosity was positively associated with Family Climate, with a small-medium effect size (f^2^ = 0.07). The latter was negatively associated with Anger Dysregulation (f^2^ = 0.07). The most robust relationship was again for Anger Dysregulation with Deviance (f^2^ = 0.93). Next, we examined possible indirect relationships of Family Religiosity with Deviance Propensity through Family Climate and Anger Dysregulation. As in single-group analysis, Family Religiosity approached a significant indirect effect with Deviance Propensity, which achieved the full significance using a one-tailed test (Indirect = −0.04; 95% CI [−0.05; −0.02]). Unlike single-group analyses, the indirect effect of Family Climate on Deviance Propensity through Anger Dysregulation turned out statically significant for religious justice-involved juveniles (Indirect = −0.17; 95% CI [−0.30; −0.06]).

## 4 Discussion

### 4.1 The influence of family religiosity and family climate in anger dysregulation and deviance propensity

Our study focused on the influence of Family Religiosity and Family Climate on Anger Dysregulation and Deviance Propensity in a sample of 214 boys, divided into two groups: religious (*n* = 112) and non-religious justice-involved juveniles (*n* = 102). According to the literature on the association between religiosity and criminality in adolescence (Salas-Wright et al., 2015; Salvatore, 2018), we identified two exogenous variables - Family Religiosity and Family Climate - and two endogenous variables – Anger Dysregulation and Deviance Propensity. Family Religiosity is based on the family’s propensity to be religious, religiously observant, and provide a religious discipline; while Family Climate was measured by considering the family support and the communication among the family members; Anger Dysregulation describes the propensity to be angry, hostile, and verbally aggressive; and Deviance Propensity is composed by deviant behavior, moral disengagement, and physical aggression.

Regarding the first hypothesis (H1) concerning the possible role of buffers of Family Religiosity and positive Family Climate for Anger Dysregulation and Deviance Propensity, in the structural model with all the samples, we found that Family Religiosity was positively associated with Family Climate that was negatively associated with Anger Dysregulation and Deviance Propensity, and Anger Dysregulation was positively related to Deviance Propensity. This result is in line with the literature (Saladino et al., 2021). Indeed, positive communication among family members leads children to develop emotional regulation skills, and family and religious values and might mediate the relationship between familial religiosity and children’s religiosity (Pickering and Vazsonyi, 2010; Guo, 2018).

Juveniles with open communication with their parents are more likely to hold religious beliefs, participate in social activities, and display pro-social behavior (Jang et al., 2018). Thus, committed religiosity seems to have a protective role in youth criminality (Li and Chow, 2015). Also, perceived support leads adolescents and young adults to follow family values instead of peer group values, especially deviant ones (Harris et al., 2017). According to Hirschi (1969) social control theory, these elements characterize social bonds and might reduce the tendency to commit crimes. Also, religiosity is an influencing factor for prosociality. Hirschi (1969) identified four elements for social bonds: (a) attachment, which describes emotional bonds (family, friends, teachers); (b) commitment and fear of punishments (sanctions if an individual commits a crime); (c) involvement, which refers to prosocial activities (sports, religious activities), and (d) belief in conventional society (laws and social norms).

Furthermore, the positive interaction between anger dysregulation and deviant propensity confirms the well-known correlation between the negative feelings of anger, hostility, verbal aggression with physical aggression, a tendency to deviant behavior, and moral disengagement. Indeed, justice-involved juveniles are more likely to express anger and hostility through verbal or physical violence and justify and minimize their criminal actions to reduce negative feelings associated with social judgment (Saladino et al., 2021). Also, youths between 18 and 25 years old report a higher rate of dangerous behaviors, such as substance use, unsafe sex, binge drinking, and criminal offending (Arnett, 1998; Arnett, 2015; Salvatore, 2017; Salvatore, 2018). Life course criminology researchers found a higher rate of violent and non-violent crimes among people aged 18–25, as confirmed by the Uniform Crime Report (UCR) (2016). Furthermore, young people of this age group in Youth Detention Centers face this stage of life according to the beliefs and values of prison. In Youth Detention Centers they are deprived of conventional development, like becoming parents, building a love relationship with a partner, and other normative life events. Mostly, juveniles explore adulthood in a punitive environment in which fellow prisoners assume a parental role (Saladino, 2020).

### 4.2 The moderating role of interiorized religiosity

Regarding the second hypothesis (H2), concerning the possible moderating role of personal religiosity in the described relationships between variables, the multigroup analysis confirmed that for justice-involved juveniles who interiorized religious discipline and beliefs, Family Religiosity showed a positive association with Family Climate, which had a negative relationship with Anger Dysregulation, which strongly predicted Deviance Propensity. Differently from the total sample, Family Climate was not directly associated with Deviance Propensity. No significant relationships between variables were found in non-religious juveniles.

This result confirmed the first hypothesis and underlying the importance for justice-involved juveniles to define themselves as religious. Indeed, according to the literature, the interiorization of religious beliefs and the professing of religion are also associated with a higher level of self-control, which reduces deviant behavior (Kadri et al., 2019). Religious bonds can provide a moral compass and lead to internal social control, the development of positive social networks, and the decrease of dangerous behaviors (Salvatore, 2018).

Our results indicated that involvement in religious practices and religious discipline within the family increases prosocial behavior among juveniles, who are less likely to engage in criminal, aggressive behavior, and other risky conduct. This evidence is also consistent with earlier studies according to which religious juveniles perceived a lower risk of externalizing behaviors such as substance use, risky sexual behavior, and delinquent activities (Chamratrithirong et al., 2013; Desmond et al., 2013). Also, interiorized religiosity is associated with high internal motivation, private beliefs, and desistance to minor and serious crimes (Salas-Wright et al., 2015).

### 4.3 Limitations

The current study presents some limitations. Firstly, it is cross-sectional, limited to a specific part of justice-involved juveniles, and not generalizable. Secondly, it was conducted in complex contexts, Youth Detention Centers, in that data could be invalidated due to the fear of exposure, and conflictual relationships with police officers and other justice-involved individuals. These reasons can lead participants to feel uncomfortable answering honestly. Third, the protocol as designed could be subjected to cognitive bias, due to the self-report and the retrospective design of measure. Fourth, we used a single-item evaluation to investigate the concept of religiosity. This method could lead to misunderstandings linked to the sensitivity of the concept studied. For all these reasons our findings should be interpreted cautiously.

## 5 Conclusion and future implications

To conclude, future studies should investigate the role of personal religiosity associated with prosocial behavior and the decrease of criminal offending; exploring differences between adolescents and emerging adults to promote desistance from crime in justice-involved and to prevent the development of deviant behavior in non-justice-involved juveniles. Also, it could be useful to conduct longitudinal studies to measure the impact of family and personal religion more rigorously on deviant behavior among juveniles. As emerged from the literature and our results, the concept of religiosity needs to be explored, especially in a high-risk sample, such as justice-involved juveniles. Religiosity could be interpreted as a protective factor that influences deviant propensity and anger in juveniles. Indeed, individuals who interiorize religious beliefs and profess religion are more likely to experience a higher level of self-control, reducing deviant behavior. Future prevention and intervention development might be based on increasing and promoting personal and familial religious bonds.

This perspective could also be considered by groups, communities, collectives, and individuals in promoting initiatives aimed at juvenile well-being. Specifically, actions could be implemented to reduce all forms of violence, including child exploitation by organized crime. For instance, in the South of Italy, many children and adolescents are involved in deviant behaviors due to organized criminality. This involvement harms their development and characterizes a social failure. Moreover, the institution should have a higher involvement and role in guaranteeing a safe environment for juveniles, often forced by criminal or abusive family backgrounds to commit illegal actions to survive or escape from abuse.

To promote these goals, communities, individuals, and social groups should change their perspective, emphasizing social rehabilitation, rather than only relinquishing the punishment of young people defined as deviant individuals, a definition which removes from the humanization and rehabilitation of the justice system. Specifically, religion could have a protective role in aggressive acts that commonly occur in correctional facilities. Religious values and beliefs could lead to prosocial attitudes toward operators and other justice-involved persons, incrementing positive relationships.

Indeed, mostly, in correctional facilities adolescents imitate negative and aggressive behaviors to integrate into the peer system, as in the social system. This tendency could be reduced by positive models such as those promoted by the community and altruistic values that religion generally professes. Unfortunately, the religious aspect is not always taken into consideration in these contexts or is underestimated in the involvement of young people. With this in mind, it would be useful for clinicians and researchers to investigate how religious activity, and the internalization of religious value associated with empathy, listening, prosociality, and altruism, impacts the behavior and well-being of justice-involved youths. Furthermore, religion could be a positive treatment tool to be encouraged by correctional facility operators.

In conclusion, it is important to pay more attention to the behavior implemented by adolescents rather than to the meaning expressed through these actions, which are not always clear and linear, but mostly symbolic and hidden. Antisocial behavior could be an expression of plight through which adolescents communicate their negative emotions and feelings such as confusion, and interpersonal difficulties, which involves a situation of suffering and discomfort and may manifest on individual, social, family, and friendship levels. The plight involves a progressive closure in themselves that implies an increase of confrontation with peer groups to belong. For the adolescent who feels a strong sense of unease, it is not the action that is covered with meaning but the underlying motivation.

As mentioned above, criminality in adolescents assumes different reasons than in adulthood. For this reason, it is important to analyze the meaning of juvenile crimes according to the developmental perspective to promote juveniles’ well-being and internalized religious values could be a positive and effective resource.

## Data availability statement

The raw data supporting the conclusions of this article will be made available by the authors, without undue reservation.

## Ethics statement

The studies involving humans were approved by University of Cassino and Southern Lazio. The studies were conducted in accordance with the local legislation and institutional requirements. Written informed consent for participation in this study was provided by the participants' legal guardians/next of kin.

## Author contributions

VS and VV: conceptualization. OM and ML: methodology, formal analysis, and data curation. OM: resources and funding acquisition. VS and OM: writing – original draft preparation. VS, OM, ML, and CC: writing – review and editing. CC, VV, and ML: supervision. VS: project administration. All authors contributed to the article and approved the submitted version.
